# Evaluation of a Mental Health Care App–Based Fatigue Management Program for Patients With Inflammatory Bowel Disease in Remission: Protocol for a Randomized Controlled Trial

**DOI:** 10.2196/96593

**Published:** 2026-09-28

**Authors:** Makoto Tanaka, Sayaka Wakai, Kayoko Sakagami, Aki Kawakami, Hikari N Takashina, Jeongae Kang, Masakazu Nagahori, Hiroaki Ito

**Affiliations:** 1 Department of Adult Health Nursing, Graduate School of Health Care Sciences, Institute of Science Tokyo, 1-5-45 Yushima, Bunkyo-ku, Tokyo, 1138519, Japan, 81 358035351; 2 Kinshukai Infusion Clinic, Osaka, Japan; 3 Awarefy Mental Research Institute, Awarefy Inc., Tokyo, Japan; 4 Health Science Research & Development Center, Institute of Science Tokyo Hospital, Tokyo, Japan

**Keywords:** cognitive behavioral therapy, fatigue, inflammatory bowel disease, internet-based intervention, psychological support, self‑care

## Abstract

**Background:**

Fatigue remains a major unresolved issue affecting the quality of life of patients with inflammatory bowel disease (IBD), even during clinical remission. Scalable psychological approaches are needed to support effective fatigue management.

**Objective:**

The aim of this study is to evaluate the effectiveness of a multicomponent mobile cognitive behavioral intervention package for fatigue management among patients with IBD in remission and to explore factors associated with engagement with the intervention and its effectiveness.

**Methods:**

This was an open-label, waitlist-controlled randomized controlled trial. Eligible participants were adults (≥18 years) diagnosed with Crohn disease or ulcerative colitis who met remission criteria and had at least mild fatigue. Participants were randomized 1:1 using stratified block randomization based on disease type, sex, and baseline fatigue severity. The intervention was a multicomponent mobile intervention package centered on a 7-week cognitive behavioral therapy–based educational course, supplemented by adjunctive support messages. The primary outcome was fatigue, measured using the Functional Assessment of Chronic Illness Therapy—Fatigue (FACIT-F), and the secondary outcome was self-efficacy, measured using the 13-item Inflammatory Bowel Disease Self-Efficacy Scale Short Form (IBD-SES13). A sample size of 175 participants per group was required.

**Results:**

Recruitment of participants began on July 28, 2025, and ended on February 24, 2026. A total of 502 patients with IBD in clinical remission agreed to participate and were assessed for eligibility. Among them, 351 met the eligibility criteria and were randomized, while 151 (30.0%) had FACIT-F scores of 40 or higher and were excluded. Currently, follow-up is ongoing. The clinical outcome data will be analyzed after follow-up is completed, and the results are expected to be submitted for publication in 2027.

**Conclusions:**

This study may contribute valuable knowledge regarding participant engagement and the potential impact of a multicomponent mobile cognitive behavioral intervention package on fatigue in patients with IBD. The findings will support future refinement of the intervention and inform large-scale evaluations and implementation efforts.

## Introduction

Inflammatory bowel disease (IBD) is a chronic inflammatory condition of the gastrointestinal tract characterized by periods of exacerbation and remission. It encompasses 2 diseases that typically manifest in early adulthood: ulcerative colitis (UC) and Crohn disease (CD). The etiologies of both conditions remain unclear, and presenting symptoms include diarrhea, abdominal pain, bloody stools, and extraintestinal manifestations in the joints and skin [1-3]. The prevalence of these 2 diseases, which significantly impact the social lives of people in their active working years, continues to rise steadily, making the establishment of support systems an urgent priority [4]. The development of effective therapeutics that enable better disease control has improved IBD management. However, previous research indicates that many patients experience difficulties in their daily lives, even during remission [5,6]. Fatigue is a common and burdensome symptom of IBD that substantially impairs health-related quality of life (QoL) and contributes to disease-related anxiety [7]. Previous studies have shown fatigue prevalence rates of 72% during active disease and 47% during remission, indicating that fatigue persists even when the disease is quiescent [8]. While the exact causes of fatigue in patients with IBD are not fully understood, factors such as sleep disturbance, anxiety, depression, and anemia have been implicated [8-10]. These factors are interrelated, with fatigue and psychological state also affecting abdominal symptoms such as abdominal pain and diarrhea, further worsening the QoL of patients with IBD. Therefore, effective strategies are needed to address fatigue as part of the overall symptom burden of IBD. Fatigue in patients with IBD has been identified as a high-priority research topic in Europe [11], with ongoing investigations into the relationship between IBD and fatigue.

A meta-analysis of nonpharmacological interventions for fatigue management in IBD revealed fatigue-reducing effects, with programs incorporating repeated face-to-face psychotherapy, particularly those based on cognitive behavioral approaches, showing strong effects [12]. However, such approaches may be difficult to implement in the routine care of working-age patients with IBD. Stress management and mental health care have long been emphasized for preventing IBD flare-ups and have now been shown to be important for managing fatigue [13]. Conversely, patients with IBD in remission typically visit the clinic only once every few months, making it difficult for them to attend in-person sessions while in school or working. Initiatives are required to enhance the efficient use of health care resources and improve patient convenience.

In recent years, digital self-management and mental health interventions have been explored as potential approaches to address fatigue and related symptoms in IBD. The IBD-BOOST program represents the largest and most rigorous evaluation to date, using a facilitator-supported, digital cognitive behavioral self-management program targeting fatigue, pain, and urgency in a multicenter, randomized controlled trial [14,15]. Although no significant effects were observed in the intention-to-treat (ITT) analysis, secondary analyses suggested potential benefits among participants who engaged in the intervention, highlighting the importance of engagement in digital cognitive behavioral self-management approaches [15,16]. In another study, a smartphone app incorporating gamification was evaluated using a nonrandomized design. Although this study did not include a control group, improvements in fatigue and related outcomes were observed among participants who completed the program, suggesting that the digital intervention may have been effective [17]. Together, these studies show that digital cognitive behavioral interventions have been explored for fatigue management in IBD but also have important limitations, including modest effects in the ITT analysis. Notably, neither the IBD-BOOST trial nor any other digital intervention study restricted its study population to patients in clinical remission. Fatigue in IBD is heterogeneous, and its underlying contributors may differ according to disease activity. During active disease, fatigue is more likely to be influenced by ongoing intestinal inflammation and its systemic consequences, whereas during remission, psychological factors, sleep disturbances, and cognitive behavioral mechanisms may play a more prominent role.

Consequently, it remains unclear whether a more targeted approach that focuses specifically on fatigue during clinical remission and that uses a general mental health smartphone app to address common psychological and cognitive behavioral mechanisms would be effective in a randomized controlled trial. The aim of this study is to evaluate the effectiveness of a multicomponent mobile cognitive behavioral intervention package for fatigue management among patients with IBD in clinical remission and to explore factors related to participant engagement that may influence its effectiveness.

## Methods

### Study Design

This study is an open-label, waitlist-controlled randomized controlled trial, developed and reported in accordance with the SPIRIT 2025 statement [18] (Checklist 1). The overall study flow is shown in Figure 1. The primary outcome comparison will be conducted between T0 and T1. Participants in the waitlist control group receive usual care during the initial study period and are offered access to the intervention after completing the primary end point assessment. Participants who meet all eligibility criteria were registered in the electronic data capture system, eACReSS, which also executes the randomization procedures. Participants were randomly assigned in a 1:1 ratio to either the intervention group or the waitlist control group. Randomization was performed using stratified block randomization within the eACReSS system, with stratification based on three factors: (1) disease type (CD or UC), (2) sex (female or male), and (3) baseline fatigue level measured using the Functional Assessment of Chronic Illness Therapy—Fatigue (FACIT-F) score (<30 or ≥30). A fixed block size of 4 was used to ensure balance across groups. The block size and randomization sequence were not disclosed to investigators or site personnel. The randomization module in the eACReSS system automatically generates and assigns allocations, and investigators cannot access future assignments; therefore, allocation concealment will be maintained. This trial is open-label; therefore, participants and study staff are not blinded to group allocation. This trial was registered with the UMIN Clinical Trials Registry on June 1, 2025 (UMIN000057278).

**Figure 1.**
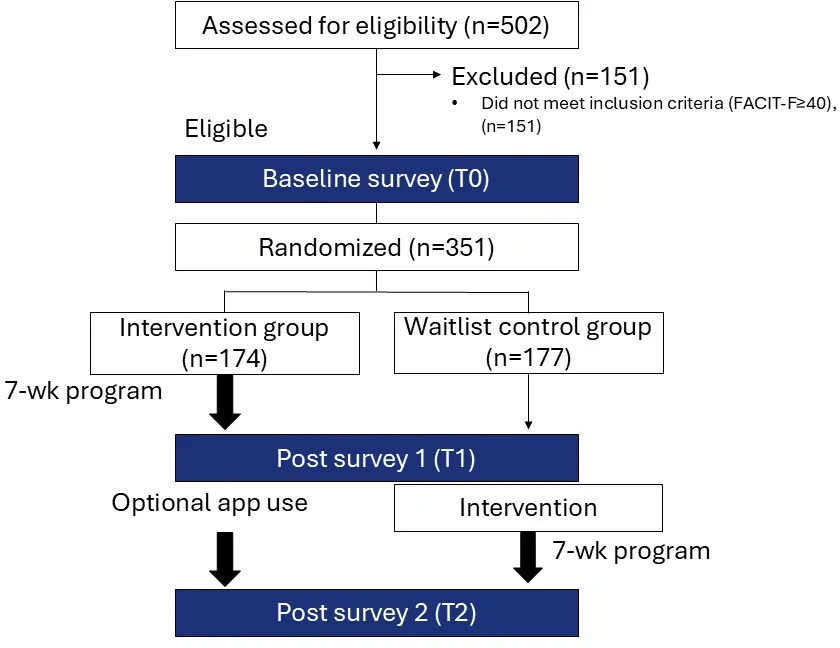
Study flow diagram. FACIT-F: Functional Assessment of Chronic Illness Therapy—Fatigue.

### Trial Setting and Participants

Participants were recruited from 2 urban, high-volume IBD care facilities in Japan: a specialized IBD clinic in Osaka and the IBD center of a university hospital in Tokyo. At both participating sites, usual care for IBD in remission typically involves routine clinical follow-up every 2 to 3 months. This care does not include systematic fatigue screening nor are written or digital educational materials or psychological guidance provided for fatigue management. The eligibility criteria for recruiting participants were as follows: adults (≥18 years) diagnosed with CD or UC who are in clinical remission, defined as a Crohn Disease Activity Index (CDAI)<150 for CD [19,20] or a partial Mayo score ≤1 for UC [21]. Additionally, participants were required to report at least mild fatigue, indicated by a FACIT-F score <40, understand Japanese, and provide written informed consent. Exclusion criteria were as follows: lack of access to a smartphone capable of running the study app, current treatment at a psychiatric or psychosomatic clinic, participation in other clinical trials, absence of a scheduled clinic visit within 2 to 5 months after the intervention period required for outcome assessment, or a judgment by the investigators that participation would be inappropriate.

During routine outpatient visits, research staff briefly introduced the study using a flyer. Individuals who expressed interest received full information and, after providing consent, were screened using the FACIT-F. Those who met eligibility criteria were registered in the eACReSS system for allocation. Recruitment continued until the target sample size was reached.

Participants will discontinue study participation if any of the following events occur:

- Withdrawal of consent at the participant’s discretion for any reason.
- Clinical deterioration or other health concerns for which the site investigator (or a delegated study clinician) judges continued participation to be inappropriate.
- Ineligibility was identified post enrollment, such that the participant no longer meets the inclusion or exclusion criteria.
- Early termination of the trial (eg, study suspension or closure by the sponsor or the institutional review board).
- Inability to continue follow-up within the study framework (eg, relocation or other circumstances that preclude scheduled assessments).
- Any other reason for which the investigator determines that continued participation has become impracticable or is not in the participant’s best interests.

If a participant discontinues after randomization, including for nonmedical or technical reasons, no further patient-reported outcome data are collected. These cases are documented as postrandomization withdrawals and handled as missing outcome data according to the prespecified statistical analysis plan (SAP). No interim analyses are planned, and no stopping guidelines have been specified.

To reduce study-related burden and compensate participants for their time, an electronic gift voucher of JP¥ 500 (approximately US $3.33) is provided by email at each of the 3 study visits (T0, T1, and T2), totaling JP¥ 1500 (approximately US $10.00) per participant for full participation. This compensation plan was reviewed and approved by the respective institutional ethics committees.

### Interventions

The intervention is designed as a multicomponent mobile intervention package consisting of an app-based program and adjunctive support messages. The primary platform is the Awarefy app, a commercially available nonmedical self-care app for mental health that includes an integrated suite of cognitive behavioral therapy (CBT)–based tools. The Awarefy app is provided in its production version (versions 23.1.7 through 24.10.5; build dates June 6, 2025, and May 28, 2026). Participants use the current version available in app stores, with standard auto-updates permitted. We confirmed that these routine maintenance updates did not modify the CBT course content or core functionalities used in this study. The app supports iOS 15.8 or later (Apple Inc) and Android 10.0 or later (Google LLC). Participants who cannot install the app were deemed ineligible. For this study, participants are provided with access to a structured 7-week CBT-based educational course titled “Skills for Managing Fatigue.” Prior to the trial, this study-specific educational course was developed based on core principles of CBT, for which there is substantial evidence supporting its effectiveness in fatigue management across various chronic conditions [22,23]. Core components of the intervention include behavioral activation, activity pacing, cognitive restructuring targeting unhelpful illness beliefs, and sleep-related strategies, which were selected based on evidence from prior meta-analytic findings in chronic fatigue populations and are considered to address factors that maintain fatigue. The app itself is not IBD-specific and is available to the general public. For this study, we collaborated with the developer to create a new educational course on fatigue management. Although developed for this trial, the course is available to all app users. The course content was developed by a team of clinical psychologists affiliated with the app developer, including 2 of the authors—one with expertise in cognitive behavioral approaches for somatic symptoms and another specializing in digital cognitive behavioral interventions—together with input from the study team. The intervention components and delivery format were informed by a pilot phase involving 11 patients with IBD. These individuals used specific features of the app and completed a questionnaire regarding its ease of use, their preferences, and its usefulness. Based on their input, specifically regarding the difficulty of maintaining a digital self-management habit, the study team integrated automated supportive messages to complement the core app-based course. This refinement aimed to enhance engagement with and adherence to the intervention within the clinical IBD population.

Participants were instructed to download the Awarefy app and were asked to complete the 7-week course titled “Skills for Managing Fatigue,” which includes the skill components summarized in Table 1, with details shown in Table S1 and Figure S1 in Multimedia Appendix 1. While the course provides the primary educational framework, the app’s integrated tools—including mood and activity logs, guided audio exercises, and a generative AI–based chat tool—provide additional practical resources for applying the cognitive behavioral skills learned in each chapter in real-world settings. No individualized feedback or therapist contact is provided as part of the intervention.

**Table 1.** Components of the 7-week “Skills for Managing Fatigue” course.

Chapter number: primary component	Core content	Hypothesized mechanism
Chapter 1: Psychoeducation	Program overview; biopsychosocial factors related to fatigue; cognitive behavioral model of fatigue; maintenance (vicious cycle) model; introduction to self-monitoring (sleep diary, activity logs)	Enhancing understanding of fatigue; increasing awareness of behavioral and cognitive patterns maintaining fatigue
Chapter 2: Activity Pattern Assessment	Assessment of activity balance (overactivity vs underactivity), including activity-type classification; identification of individual-activity patterns	Identifying maladaptive behavioral patterns and imbalance in activity contributing to fatigue
Chapter 3: Behavioral Activation	Goal setting; activity scheduling; graded engagement; balancing activity and rest (including pacing)	Establishing adaptive activity patterns; optimizing activity-rest balance and energy regulation
Chapter 4: Mindfulness	Attention training to reduce excessive focus on fatigue; mindfulness practice	Reducing attentional bias toward fatigue; decreasing excessive symptom monitoring
Chapter 5: Cognitive Restructuring	Cognitive restructuring targeting fatigue-related beliefs	Modifying maladaptive cognitions (eg, catastrophic interpretations); improving cognitive flexibility
Chapter 6: Review and Maintenance	Review of skills; maintenance planning; relapse prevention	Consolidating learned skills; promoting long-term self-management
Chapter 7: (optional) Sleep Intervention	Sleep hygiene education; sleep restriction; stimulus control	Improving sleep quality; reducing physiological vulnerability to fatigue

In addition to the app-based course, participants receive automated reminder messages through a separate messaging app. These messages include brief texts and short videos on factors associated with fatigue in IBD and daily self-management strategies, primarily intended to support engagement with the app-based intervention.

### Outcomes

#### Primary Outcome

The primary outcome is fatigue, measured using the FACIT-F [24]. This scale ranges from 0 to 52, with higher scores indicating less fatigue and better QoL. Several instruments have been used to assess fatigue in IBD; however, no single instrument is universally accepted as a standard. Among available measures, the Multidimensional Fatigue Inventory [25] and the FACIT-F scale are most frequently used in IBD research [8,9]. The FACIT-F is brief, easy to complete, and has demonstrated strong reliability and validity in both CD and UC [26]. Additionally, its interpretability has been confirmed in recent trials, and the minimal clinically important difference for the FACIT-F score has been reported to be approximately 3 to 4 points [27,28]. Because the FACIT-F is widely used internationally, results can be compared across studies and with data from the general population and other chronic conditions. Given these advantages, the FACIT-F is used to measure fatigue as a primary outcome in this trial. Informed by prior literature [26], a FACIT-F score <40 was used to identify participants with at least mild-to-moderate fatigue, while a cutoff of 30 (<30 or ≥30) was used for stratification to distinguish clinically relevant levels of fatigue.

#### Secondary Outcome

The secondary outcome is self-efficacy for IBD self-management, measured using the 13-item Inflammatory Bowel Disease Self-Efficacy Scale Short Form (IBD-SES13) [29]. Self-efficacy represents an individual’s belief in their capacity to adopt the behaviors required to manage a disease. The IBD-SES was developed to evaluate IBD-specific self-efficacy and has demonstrated psychometric properties associated with psychological distress and a moderate correlation with QoL. The IBD-SES13 comprises 13 items in four domains: (1) managing stress and emotions, (2) managing medical care, (3) managing symptoms and disease, and (4) maintaining remission. Scores for each item range from 1 (“not at all”) to 10 (“totally sure”), with higher scores indicating greater self-efficacy [29].

#### Exploratory Outcomes

Exploratory outcomes include self-reported daily sleep duration and emotional well-being using the Emotional Well-Being (EWB) subscale of the FACIT and Functional Assessment of Cancer Therapy (FACT) measurement system [24]. The EWB is a 6-item patient-reported subscale (range 0‐24), with higher scores indicating better emotional well-being. It is part of the core Functional Assessment of Cancer Therapy–General (FACT-G) domains used in the FACIT system and is available in multiple validated translations. The EWB subscale is included to capture the psychological dimension related to fatigue, such as mood and affective burden, that may change alongside fatigue severity (FACIT-F), thereby complementing the primary outcome.

### Assessment and Participant Timeline

#### Time Points

Table 2 shows the assessment and participant timeline. Assessments will be conducted during routine, face-to-face outpatient clinic visits. Baseline assessment (T0) is performed at enrollment. The first follow-up assessment (T1) is conducted at the next scheduled clinic visit, which is approximately 8 weeks after baseline but may vary according to individual visit schedules. The second follow-up assessment (T2) is conducted at the subsequent clinic visit, typically 2 to 3 months after T1.

**Table 2.** Participant timeline.

	Enrollment		Postrandomization	
	Eligibility screen (–T)	Baseline (T0)	T1	T2
Randomization		✓		
Assessment				
Clinical characteristics^a^		✓		
Sociodemographic data^b^		✓		
FACIT-F^b,c^ (Fatigue scale)	✓	✓^d^	✓	✓
IBD-SES13^b,e^		✓	✓	✓
FACIT^b^ (EWB^f^ subscale)		✓	✓	✓
Sleep duration^b^		✓	✓	✓
Self-report app use^b^		✓	✓	✓
Disease activity^a^ (CDAI^g^/partial Mayo)	✓	✓^d^	✓	✓
Laboratory data^a^ (CRP^h^, Hb^i^, Alb^j^)		—^k^	—^k^	—^k^
Awarefy app-usage logs^l^			✓	✓

a Extracted from medical records (routine clinical documentation).

b Collected via self‑administered questionnaire.

c FACIT-F: Functional Assessment of Chronic Illness Therapy—Fatigue.

d T0 values were carried forward from the eligibility assessment.

e IBD-SES13: 13-item Inflammatory Bowel Disease Self‑Efficacy Scale Short Form.

f EWB: Emotional Well-Being.

g CDAI: Crohn Disease Activity Index.

h CRP: C-reactive protein.

i Hb: hemoglobin.

j Alb: albumin.

k Obtained only if measured in routine clinical care; no protocol‑mandated blood tests.

l Provided by the app developer.

#### Nonprimary Assessments

In addition to the outcomes, the following information is collected via a self-administered questionnaire: sociodemographic data (age, marital status, employment, and disease duration) and self-reported app use, captured at each study visit using a brief questionnaire offering 3 response options: “not at all,” “occasionally,” or “frequently.” Clinical characteristics (disease type, current treatments, and comorbidities), disease activity (CDAI or partial Mayo as appropriate), and available routine laboratory data (C-reactive protein, hemoglobin, and albumin) are extracted from medical records. Laboratory data are extracted when available from routine care at baseline and follow-up visits; no additional visits or venipunctures are scheduled for research purposes.

#### App-Usage Logs

Usage metrics, including session counts, days of active app use (not counting simply opening the home screen), module completion, and engagement with integrated features such as the AI chat tool and audio exercises, will be provided by the app developer via a designated coinvestigator (HNT) under a preagreed data-sharing arrangement. These data will be reported to characterize patterns of engagement with each component of the multicomponent intervention. Data containing personal identifiers or message or content are encrypted and will not be shared with the academic team. Participants will self-link their study participation by entering their research ID and in-app user code into a secure study form. This form was built and managed by a coinvestigator (HNT) to facilitate secure linkage and transfer of deidentified app-log data. The academic dataset will contain only study IDs and usage variables, with no direct identifiers. App-usage logs will be extracted by Awarefy engineers and transferred to the research team (MT and SW), who will link these with the clinical outcome data for analysis.

### Statistical Analysis

#### Sample Size

Based on prior data showing a mean difference of approximately 5 points (corresponding to an effect size of 0.58) on the FACIT-F between patients in remission and those with active disease [10], we anticipated that the intervention would produce a more conservative effect than the difference observed between disease activity states. Therefore, assuming a standardized effect size of 0.30 (corresponding to an approximate 3-point difference in the FACIT-F score, which falls within the reported minimal clinically important difference range [27,28]), a 2-sided α of .05, and 80% power, the required sample size was estimated at 175 participants per group for a 2-arm comparison.

#### Primary Analysis

The primary estimand is the between-group difference in the adjusted mean FACIT-F score at T1 among all randomized participants, regardless of adherence to the intervention (treatment policy estimand). The primary between-group comparison at T1 will follow the ITT principle and use analysis of covariance, with treatment group as the main factor and baseline outcome as a covariate. Stratification factors (disease type, sex, and baseline fatigue level [<30 or ≥30]) will be included as fixed effects. Multiple imputation by chained equations (MICE) will be used to handle missing outcome data prior to the primary analysis. The imputation model will include baseline and other prespecified prognostic variables. Twenty imputed datasets will be generated and analyzed separately, with results pooled according to Rubin rules [30]. Further details are provided in the SAP (Multimedia Appendix 2). The SAP was finalized on July 31, 2026, prior to the database lock and before any outcome analyses were conducted.

#### Sensitivity Analysis

Participant withdrawal after randomization may result in missing post-baseline patient-reported outcome data; such missingness will be addressed using prespecified analytical approaches. A mixed model for repeated measures will be conducted using restricted maximum likelihood estimation. The model will include treatment group, visit, treatment-by-visit interaction, and baseline FACIT-F score as fixed effects. An unstructured covariance matrix will be used to account for within-participant correlations across post-baseline visits, and degrees of freedom will be estimated using the Kenward-Roger method [31] under the missing at random assumption.

A per-protocol (adherent) subset will be predefined. The adherent subset will be defined as participants who (1) completed the first 2 chapters (Chapter 1: Psychoeducation and Chapter 2: Activity Pattern Assessment) of the “Skills for Managing Fatigue” course and (2) engaged with any of the app’s core features (eg, mood and activity logs, audio exercises, AI chat, or learning modules) on at least 1 day per week for ≥5 of the 7 weeks during the intervention period. Chapter 2 was selected as the adherence threshold because, by this point, participants would have received the core psychoeducation and identified their individual fatigue patterns and corresponding coping strategies. The self-management tools introduced in Chapter 1 or 2 (eg, sleep diary, mood and activity logs, goal setting, and audio-guided exercises) are independently accessible within the app, allowing participants to continue practicing CBT skills without completing all subsequent learning modules. This definition aims to capture active engagement with the therapeutic components of the intervention, distinguishing purposeful use from merely accessing the app. Further sensitivity analyses planned for this study are provided in the SAP (Multimedia Appendix 2).

#### Exploratory Analysis

Descriptive trajectories at T0, T1, and T2 will be presented to illustrate within-group patterns. No between-group inference will be performed at T2.

The trends in engagement indicators—including self-reported app-use frequency and app-usage logs—will be described over the intervention period. The association between improvements in FACIT-F scores and app-usage logs will also be examined. In addition, trajectories of FACIT-F, IBD-SES13, and EWB will be compared to assess whether improvement in fatigue is accompanied by changes in self-efficacy and emotional well-being. Factors potentially related to intervention effectiveness will also be explored. *P* values from exploratory analyses will not be adjusted for multiple comparisons and will be interpreted as hypothesis-generating rather than confirmatory.

### Study Governance

The primary statistical analysis will be conducted by the lead academic investigator (MT), and Awarefy employees (HNT and JK) will not be involved in the analysis. The academic team maintains full ownership and custody of the primary outcome dataset; the sponsor has access only to deidentified app-usage logs and will not have access to participant-level clinical data at any point. Awarefy Inc has no role in participant allocation, outcome assessment, statistical analyses, study-site selection, determination of eligibility criteria, or selection of study outcomes. The specific app-usage variables to be analyzed were predefined by the lead academic investigator (MT) based on the study hypotheses, and the Awarefy employee (HNT) is responsible only for the technical extraction and transfer of the requested data. Furthermore, the sponsor holds no contractual rights of veto or organizational review over the final manuscript, and no publication delays are permitted. Coauthors employed by Awarefy (HNT and JK) participated in manuscript preparation and review, particularly regarding the description of the intervention and technical aspects, in their roles as individual authors rather than as representatives of the sponsor. They had no authority over the reporting or publication of the study findings. Only the lead academic investigator (MT) and HNT from Awarefy have access to the linkage table. Participant-level linkage between research IDs and app codes will be performed by the academic investigators (MT and SW) following the transfer of usage logs from the developer.

### Ethical Considerations

This study is being conducted in accordance with the principles outlined in the Declaration of Helsinki. Ethical approval was obtained from the Ethics Review Committee of the Faculty of Medicine, Institute of Science Tokyo, and the Ethics Committee of Medical Corporation Kinshukai (approval numbers: I2025-012 and 2025‐10). Written informed consent was obtained from all participants by trained research nurses or designated study staff at each site before enrollment.

This study uses a commercially available mental health care app that includes both free and paid features. All app functions were made available at no cost to participants for the duration of the study, and access will end according to the app’s standard terms outside the study period. Participants were informed that the Awarefy app is a commercially available app that integrates tools based on CBT and was not developed specifically for IBD. Data were managed in the eACReSS system using pseudonymous study IDs; routine clinical data will be extracted from medical records when available, and no protocol-mandated blood tests will be performed. Participants will be able to withdraw at any time without any impact on usual care. To minimize potential risk, individuals currently receiving treatment at a psychiatric or psychosomatic clinic were excluded from participation. Given the low-risk nature of the intervention, no formal monitoring of trial conduct or additional compensation for harm is planned. All data entered into the app are encrypted and cannot be accessed by the research team. Therefore, no real-time monitoring of participant responses is performed. The app includes a safety disclaimer, advising users who experience distress or worsening symptoms to consult their treating physician. Information on publicly available mental health support services is also provided within the app (Figure S2 in Multimedia Appendix 1). The AI chat tool was developed in collaboration with a licensed psychologist and provides appropriate guidance to seek professional or other support when users express severe psychological distress. Participants who experience health concerns during the study will be advised to seek routine medical care as appropriate. Any serious psychological adverse events reported by participants during study contacts will be assessed for severity and potential relatedness to the intervention and reported in the final study results.

The findings will be disseminated through peer-reviewed publications and reported in the trial registry. Any important protocol modifications will require approval by the institutional ethics committee before implementation. Where applicable, the trial registry will be updated, and participants will be informed of any modifications that may affect their participation.

## Results

Recruitment of participants began on July 28, 2025, and ended on February 24, 2026. A total of 502 patients with IBD in clinical remission agreed to participate and were assessed for eligibility in the study. Among them, 351 met the eligibility criteria and were randomized, while 151 (30%) had FACIT-F scores of 40 or higher and were excluded. Recruitment continued until the target sample size was reached. Because recruitment was monitored on a daily basis rather than being automatically terminated by the electronic data capture system, one additional participant was enrolled before recruitment was closed, resulting in a total of 351 participants. Participant follow-up is expected to conclude in late August 2026. This manuscript reports the finalized study protocol and current trial status. Following database lock, approximately 4 to 6 months will be required for data cleaning, linkage of app-usage logs, and statistical analyses. The primary results are therefore expected to be submitted for publication in 2027.

## Discussion

This protocol describes a pragmatic randomized controlled trial designed to evaluate the short-term effectiveness of a multicomponent mobile cognitive behavioral intervention package for fatigue management and to explore factors associated with engagement and treatment response among individuals with IBD experiencing fatigue. Fatigue remains one of the most burdensome symptoms for people with IBD, even during periods of clinical remission; however, effective and scalable self-management strategies are limited [7]. By incorporating validated patient-reported outcome measures alongside real-world app-usage data, this trial aims to generate evidence not only on clinical effectiveness but also on how participants engage with the program in routine outpatient care.

A key strength of this study is its pragmatic design, which aligns assessments with routine clinic visits and minimizes participant burden. The use of standardized instruments, including the FACIT-F and IBD-SES13, facilitates the interpretation of intervention-related changes in fatigue and self-efficacy in relation to existing IBD literature, while deidentified app-usage logs enable a detailed characterization of engagement patterns. This combination provides an opportunity to examine how engagement with digital interventions relates to changes in fatigue and related psychological constructs under real-world conditions.

Previous digital interventions for IBD, such as the IBD-BOOST program, have targeted multiple co-occurring symptoms, including fatigue, pain, and urgency, reflecting the complex and interrelated symptom burdens experienced by patients with IBD [14,15]. Although the IBD-BOOST trial evaluated a facilitator-supported digital CBT program, it did not demonstrate a significant ITT effect. One possible explanation is that the trial included patients with both active disease and remission, whereas CBT-based self-management strategies may be more effective when symptoms are relatively stable and patients can engage consistently with behavioral techniques.

In contrast, this study focuses on fatigue during clinical remission, a period when ongoing intestinal inflammation is less prominent and psychological, cognitive behavioral, and sleep-related factors may play a more substantial role. By restricting the study population to patients in remission and targeting fatigue as the primary outcome, this trial seeks to evaluate a more focused intervention strategy for a clearly defined patient subgroup. In addition, while IBD-BOOST combined digital content with facilitator support, our intervention was designed as a fully mobile-based program that participants can access flexibly and at their own pace. The app also incorporated practical support for skill implementation, such as audio-guided exercises and self-monitoring tools, to facilitate the application of CBT-based strategies in daily life and promote sustained engagement.

Another important feature of this study is the evaluation of a general, non–IBD-specific mental health platform as the basis for the intervention. Fatigue is a common and persistent symptom across many chronic diseases, and interventions addressing shared psychological and cognitive behavioral mechanisms may have broader applicability beyond a single-disease context. This study will examine the potential scalability of such an approach by evaluating a general mental health care app among patients with IBD. To ensure clinical relevance for IBD, adjunctive IBD-specific reminder messages will be provided to support engagement with the intervention and to offer disease-contextualized self-management guidance.

The assumed standardized effect size of 0.30 may appear optimistic in light of the null ITT findings for fatigue in the most recent IBD-BOOST trial [15]. However, as discussed above, this trial differs from IBD-BOOST in several respects. These design features were hypothesized to improve engagement and effectiveness. Furthermore, a meta-analysis showed a pooled standardized mean difference of 0.33 for nonpharmacological interventions for IBD-related fatigue [12]. Since the effectiveness of interventions varies from study to study, the planned sample size was determined to detect an effect size considered clinically meaningful and feasible at the time the study design was established.

Several limitations should be acknowledged. First, regarding measurement and design, the open-label design and reliance on self-reported outcomes may introduce reporting bias due to positive expectancy in the intervention group or nocebo effects in the waitlist group. The use of an attention-matched comparator would have provided a more rigorous assessment of the intervention by controlling for nonspecific effects such as attention and expectancy. Future confirmatory trials should consider incorporating such a comparator. Furthermore, the waitlist control design restricts between-group comparisons to the short-term period. Clinical remission was defined using symptom-based indices rather than objective biomarkers or endoscopic findings, which may overlap with fatigue scores. Additionally, the nature of “usual care” may vary between the participating clinical sites. Second, regarding generalizability, participants were recruited from a university hospital and a specialized IBD clinic, both located in major urban areas in Japan. Although this represents different levels of specialized care, the findings may not be fully representative of patients in rural areas or those managed in general primary care settings. Third, because the intervention will require the use of a mobile app, patients with limited access to or familiarity with digital technology may be unable to participate, potentially introducing selection bias. In addition, engagement with digital interventions is inherently variable, and exploratory analyses of adherence and usage patterns will be necessary to interpret the findings. Finally, the involvement of the app developer as a study sponsor should also be considered, although safeguards were implemented to ensure the independence of the academic team in data analysis. Nevertheless, this pragmatic trial is designed to address important gaps in the current evidence base and to inform the future development and evaluation of scalable digital interventions for fatigue management in IBD.

Despite these constraints, the study is expected to contribute valuable knowledge regarding participant engagement and the potential impact of the multicomponent intervention on fatigue in patients with IBD. The findings will support future refinement of the intervention and inform large-scale evaluations and implementation efforts.

## Supplementary material

10.2196/96593 Multimedia Appendix 1 Detailed content of the “Skills for Managing Fatigue” course and representative screenshots of the Awarefy app including the safety disclaimer.

10.2196/96593 Multimedia Appendix 2 Statistical analysis plan for the randomized controlled trial.

10.2196/96593 Checklist 1 SPIRIT checklist.

## Abbreviations

CBT: cognitive behavioral therapy
CD: Crohn disease
CDAI: Crohn Disease Activity Index
EWB: Emotional Well-Being
FACIT-F: Functional Assessment of Chronic Illness Therapy—Fatigue
FACT: Functional Assessment of Cancer Therapy
FACT-G: Functional Assessment of Cancer Therapy—General
IBD: inflammatory bowel disease
IBD-SES13: 13-item Inflammatory Bowel Disease Self-Efficacy Scale Short Form
ITT: intention-to-treat
MICE: multiple imputation by chained equations
QoL: quality of life
SAP: statistical analysis plan
UC: ulcerative colitis
