# Supplementary material for: Evaluation of a Mental Health Care App–Based Fatigue Management Program for Patients With Inflammatory Bowel Disease in Remission: Protocol for a Randomized Controlled Trial
Source: JMIR Res Protoc. 2026 Sep 28;15:e96593. doi: 10.2196/96593 (PMC13618207; doi:10.2196/96593)
Supplement: Supplementary file 1 [file resprot-v15-e96593-s001.pdf]

Supplementary Table S1. Detailed Content of the 'Skills for managing fatigue' Course

Chapter	#	*Session title*	*Interactive components**
1 (5 sessions)	1	For those troubled by fatigue	8
	2	Understanding the fatigue cycle	
	3	Long- and short-term perspectives	
	4	Starting to record	
	5	Chapter review	
2 (5 sessions)	6	Your activity type	3
	7	Regulating your activity rhythm	
	8	Mood and behavior: behavioral activation	
	9	Finding enjoyable (pleasant) activities	
	10	Chapter review	
3 (5 sessions)	11	Values-based goals	10
	12	Setting your goals	
	13	Type-tailored activity–rest balance	
	14	Beginning pacing	
	15	Recording pacing & chapter review	
4 (5 sessions)	16	Fatigue and attention	8
	17	Noticing mind-wandering	
	18	Training attention-switching	
	19	Returning to the present from anxiety	
	20	Chapter summary	
5 (4 sessions)	21	Fatigue–emotion–thought–behavior links	8
	22	Catching thoughts	
	23	Acquiring multiple perspectives	
	24	Chapter summary & review	
6 (4 sessions)	25	Course summary	11
	26	High-risk situations and coping	
	27	Recovering after fatigue returns	
	28	Building supportive resources	
7 (5 sessions)	29	The vicious cycle of insomnia	1
	30	Sleep and lifestyle	
	31	Coping with sleepless nights	
	32	Reading the sleep record	
	33	Chapter summary	

*Interactive components include self-assessments; structured self-monitoring (sleep diary, daytime- mood/energy log); behavioral activation exercises; values clarification, goal setting, and action planning; activity-pacing plans and reviews; audio-guided mindfulness practices; three- and seven-column thought records; and guided reflective prompts. The count of interactive components refers to those that appear within the course. These components can also be employed independently of the educational course.

<**Supplementary Figure S1.** >

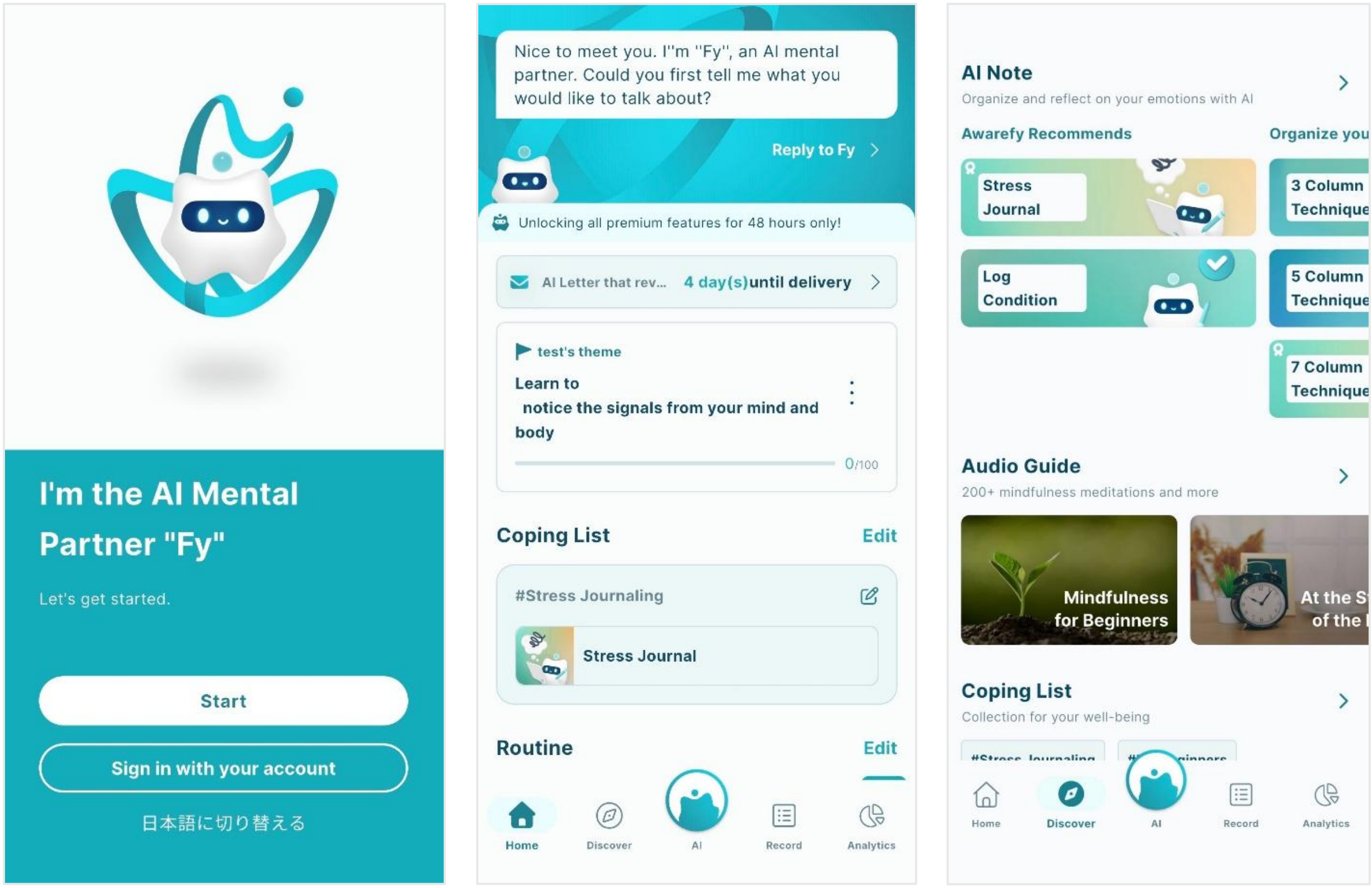

(A) Registration screen; (B) Home screen; (C) Explore screen;

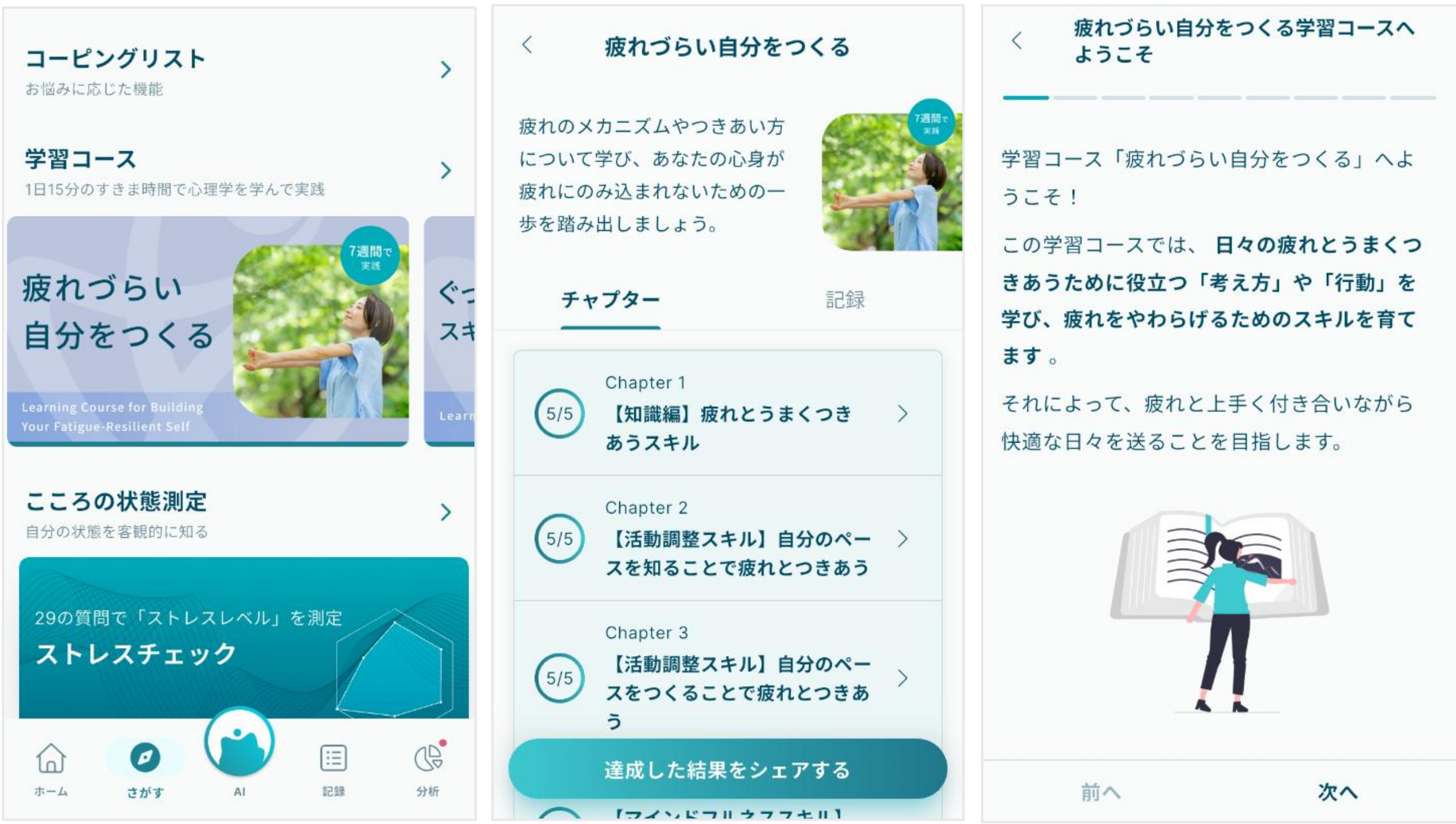

(D) Entry page of the *Skills for Managing Fatigue* course; (E–F) Representative pages from the course

Supplementary Figure S1. Representative screenshots of the Awarefy application used in the intervention (Screenshots were captured on 15 July 2026 using app version 24.10.18). ©2026 Awarefy Inc. Reprinted with permission.

The upper panels (A,B,C) show general application functions, including the registration screen, Home screen (e.g., AI Chat and Coping List), and Explore screen (e.g., Audio Guide). The lower panels (D,E,F) show representative screenshots of the CBT-based Skills for Managing Fatigue course used in this trial. The intervention course was delivered entirely in Japanese. Some general application functions also support an English user interface, and representative screenshots of these functions are shown.

<Supplementary Figure S2. >

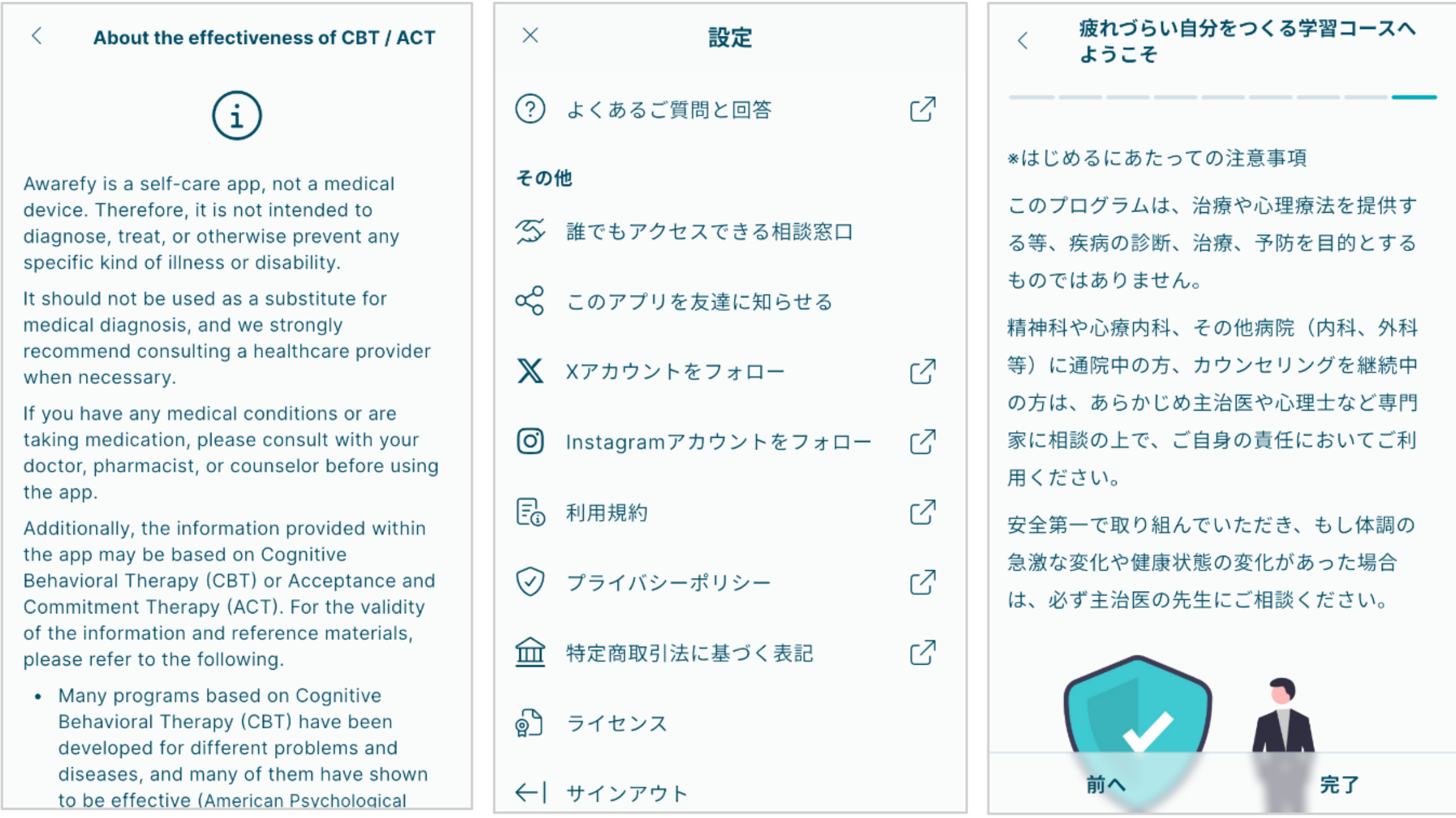

(A) General application guidance (B) Setting screen; C) Safety disclaimer at the course

Supplementary Figure S2. Screenshots of safety disclaimer in the Awarefy application (Screenshots were captured on 28 July 2026 using app version 24.10.18). ©2026 Awarefy Inc. Reprinted with permission.

The panel (A) shows general application guidance. The panel (B) shows settings screen, including a list of publicly available mental health helplines and crisis contact information accessible to all users (Japanese interface only). The panel (C) shows the safety disclaimer screenshot of the CBT-based Skills for Managing Fatigue course used in this trial. The intervention course was delivered entirely in Japanese. Some general application functions also support an English user interface, and general application guidance screenshot is shown.
