# Supplementary material for: Evaluation of a Mental Health Care App–Based Fatigue Management Program for Patients With Inflammatory Bowel Disease in Remission: Protocol for a Randomized Controlled Trial
Source: JMIR Res Protoc. 2026 Sep 28;15:e96593. doi: 10.2196/96593 (PMC13618207; doi:10.2196/96593)
Supplement: Supplementary file 2 [file resprot-v15-e96593-s002.pdf]

Supplementary Appendix 1

Statistical Analysis Plan (SAP)

Study Title: Evaluation of a mental health care app-based fatigue management program for patients with inflammatory bowel disease in remission: A randomized controlled trial protocol

Version: 1.0

Date: 31 July 2026

Author: Makoto Tanaka

Statistical Software: IBM SPSS Statistics for Windows, Version 29 (Japanese version)

1. Introduction

This Statistical Analysis Plan (SAP) provides additional details regarding the pre-specified statistical analyses described in the study protocol. This document was finalized before database lock and prior to outcome analysis and should be read in conjunction with the trial protocol.

2. Outcomes

Assessment and participant timeline

	Enrolment		Post-randomization	
**TIMEPOINT**	**Eligibility screen (-T)**	**Baseline (T0)**	**T1**	**T2**
**Randomization**		✓		
**Assessment**				
Clinical characteristics ‡		✓		
Sociodemographic data †		✓		
FACIT-F (Fatigue scale) †	✓	✓*	✓	✓
IBD-SES13 †		✓	✓	✓
FACIT (EWB subscale) †		✓	✓	✓
Sleep duration †		✓	✓	✓
Self-report app use †		✓	✓	✓
Disease activity ‡ (CDAI/partial Mayo)	✓	✓*	✓	✓
Laboratory data ‡ (CRP, Hb, Alb)		△	△	△
Awarefy app usage logs §			✓	✓

FACIT-F = Functional Assessment of Chronic Illness Therapy–Fatigue; IBD-SES13 = Inflammatory Bowel Disease Self-Efficacy Scale Short Form; EWB = emotional well-being; CDAI = Crohn's Disease Activity Index; CRP = C-reactive protein; Hb = hemoglobin; Alb = albumin

† = Collected via self-administered questionnaire
‡ = Extracted from medical records (routine clinical documentation)
§ = Provided by the app developer
* = T0 values were carried forward from the eligibility assessment.
△ = obtained only if measured in routine clinical care; no protocol-mandated blood tests

Primary outcome: FACIT-F score at T1.
Secondary outcome: IBD-SES13.
Exploratory outcomes: EWB, sleep duration, and engagement-related measures.

3. Analysis Populations

Intention-to-Treat (ITT) Population:
All randomized participants will be analyzed according to their randomized allocation regardless of adherence.

Per-Protocol Population:
Per-protocol analyses will include participants in the intervention group who met the predefined adherence criteria and participants in the waitlist control group who completed the T1 assessment.

4. Analysis Methods

4.1 General Statistical Principles

All tests will be two-sided with $\alpha=0.05$. Effect estimates will be reported with 95% confidence intervals and p-values. Baseline characteristics will be summarized descriptively. No significance testing of baseline differences will be performed.

4.2 Data Management and Variable Derivation

The final analysis dataset will be locked following completion of data collection, data integration, data cleaning, and quality checks. Electronic questionnaires require responses to all items; therefore, item-level missing data are not expected for FACIT-F, IBD-SES13, or EWB. Questionnaire scores will be calculated according to published scoring procedures. Derived variables will include FACIT-F, IBD-SES13, EWB, and engagement metrics derived from app usage logs.

4.3 Primary Analysis

The primary estimand is the between-group difference in adjusted mean FACIT-F score at T1 among all randomized participants (treatment-policy estimand). The primary analysis will compare the intervention and waitlist control groups at T1 using analysis of covariance (ANCOVA), with treatment group as the main factor and baseline FACIT-F score as a covariate. Disease type, sex, and baseline fatigue level (<30 vs ≥30) will be included as fixed effects. Multiple imputation by chained equations (MICE) will be used to handle missing outcome data prior to the primary analysis.

Distributional assumptions of the FACIT-F scores will be evaluated. If substantial violations of normality or severe floor or ceiling effects are observed, a rank-based ANCOVA will be conducted as a sensitivity analysis.

4.4 Missing Data

MICE will be performed. The imputation model will include treatment group, baseline FACIT-F score, disease type, sex, fatigue level (<30 vs ≥30), age, employment status, and the number of active app-use days. Participants who discontinued before app installation will be assigned 0 active app-use days. Twenty imputed datasets will be generated using

predictive mean matching. Results from these datasets will be pooled according to Rubin's rules. A fixed random seed of 1234 will be used to enhance the reproducibility of the imputation procedure. The extent and pattern of missing data will be summarized descriptively. The primary analysis will be conducted using multiply imputed datasets. Unless otherwise specified, sensitivity and exploratory analyses will be conducted using available data.

4.5 Sensitivity Analyses

4.5.1 Mixed Model for Repeated Measures (MMRM)

An MMRM will be fitted using restricted maximum likelihood estimation under the missing at random (MAR) assumption. The model will include treatment group, visit (T1 and T2), treatment-by-visit interaction, and baseline FACIT-F score as fixed effects. An unstructured covariance matrix will be used to model within-participant correlations across post-baseline visits, and degrees of freedom will be estimated using the Kenward-Roger method. The primary estimand from the MMRM will be the adjusted between-group difference in FACIT-F score at T1.

4.5.2 Per-Protocol Analysis

Per-protocol analysis will be performed using the predefined adherent population.

4.5.3 Site-Adjusted Analysis

The primary ANCOVA model will be repeated with study site (Osaka vs Tokyo) included as an additional covariate to assess the robustness of the findings to potential heterogeneity between clinical settings.

4.5.4 High-Engagement Analysis

The primary ANCOVA model will be repeated among participants meeting the prespecified high-engagement criteria. High-engagement participants are defined as participants who completed at least Chapter 5 and engaged with at least one core app feature on at least one day per week for ≥5 of the 7 intervention weeks. Chapter 5 was selected because participants reaching this stage have been exposed to the core CBT-based fatigue-management components of the intervention.

4.6 Exploratory Analyses

4.6.1 Outcome trajectories

Descriptive trajectories of FACIT-F, IBD-SES13, and EWB will be presented at T0, T1, and T2.

4.6.2 Sub-group analyses

Exploratory subgroup analyses will be conducted for the following baseline factors:

- Disease type (Crohn's disease vs. ulcerative colitis)
- Baseline fatigue level (FACIT-F <30 vs ≥30)
- Sex (female vs male)

・Employment status (employed vs. not employed)
For each subgroup, a separate ANCOVA model including treatment, subgroup, and a treatment-by-subgroup interaction term will be fitted. Results will be presented using forest plots with corresponding treatment-effect estimates, 95% confidence intervals, and interaction P-values. No adjustment for multiple comparisons will be performed. All subgroup analyses will be regarded as exploratory and hypothesis-generating and will be interpreted cautiously.

4.6.3 Dose-response and predictors of effectiveness

Exploratory dose-response analyses will be conducted to examine the relationship between intervention engagement and FACIT-F outcomes. Engagement measures will include the following: (1) number of active app-use days; and (2) self-reported app-use frequency. For analyses using engagement exposure as the explanatory variable, waitlist-control participants and intervention participants with no app use will be assigned an engagement value of zero. Associations between engagement exposure and FACIT-F scores at T1 will be examined using linear regression models adjusted for baseline FACIT-F scores. All exploratory analyses will be considered hypothesis-generating.

4.6.4 Engagement patterns and predictors of engagement

Intervention engagement will be summarized descriptively using app-log data. Descriptive measures will include:
・Number of active app-use days
・Number of days using the AI-based chat tool
・Number of days using audio-guided exercises
・Number of days using mood/activity logs
・Weekly app-use patterns during the intervention period
Weekly engagement trends will be presented descriptively to characterize intervention uptake, continued use, and disengagement over time. Baseline characteristics associated with greater intervention engagement, as measured by app-usage metrics, will be explored using regression-based analyses. Potential predictors will include baseline demographic factors (age, sex, marital status, and employment status), clinical factors (disease type, disease duration, and baseline fatigue level), and patient-reported measures (IBD-SES13, EWB, and sleep duration). All analyses in this section will be considered hypothesis-generating.

4.7 Statistical Software

All statistical analyses will be performed using IBM SPSS Statistics for Windows, Version 29 (Japanese version).

4.8 Deviations from SAP

Any deviations from this SAP will be documented, justified, and reported in subsequent publications.
